# Knowledge, attitude, and perception towards COVID-19 vaccinations among the adults in Rwanda: a cross-sectional study

**DOI:** 10.1186/s12889-024-19082-9

**Published:** 2024-07-17

**Authors:** Abakundana Nsenga Ariston Gabriel, Xiao-Yang Wang, Laila Jamil, Mulugeta Shegaze Shimbre, Gerard Bikorimana, Lin Zhao, Wu-Chun Cao

**Affiliations:** 1 Institute of EcoHealth, School of Public Health, Cheeloo College of Medicine, Shandong University, Jinan, P. R. China; 2 Department of Epidemiology, School of Public Health, Cheeloo College of Medicine, Shandong University, Jinan, P. R. China; 3 School of Governance, Development and Society, College of Arts and Social Sciences,University of Rwanda, Kigali, Rwanda; 4 State Key Laboratory of Pathogen and Biosecurity, Beijing Institute of Microbiology and Epidemiology, Beijing, P. R. China

**Keywords:** Attitudes, Health beliefs, Knowledge, Perception, Vaccination, Vaccine, Rwanda

## Abstract

**Background:**

Multiple vaccinations have received approval for the prevention of the coronavirus illness. Nevertheless, the sluggish vaccination rate is mostly attributed to the general population’s limited understanding and unwillingness to accept the use of vaccinations. Thus, it is important to investigate the Rwandan population’s knowledge, attitudes, and perceptions toward COVID-19 vaccines.

**Methods:**

A cross-sectional survey was conducted among 370 participants from 11th to 17th February 2023. Demographic information was gathered, and knowledge, attitudes, and perceptions of COVID-19 vaccinations were assessed. A binary logistic regression analysis was undertaken to determine the parameters that determine the perception of COVID-19 vaccinations.

**Results:**

This study included 370 participants. Among them, 85% had good knowledge about COVID-19 vaccines, and 84% had a positive attitude towards them. Additionally, the study had a diverse group, with half of the participants being female and nearly half falling between the ages of 30 and 39. Several key findings emerged through logistic regression analysis. Those aged 30–39 had 1.39 times higher odds of positive perception than 18–28 (OR = 1.39, 95% CI = 1.08–3.24). Participants with a university education were twice as likely to have a positive perception compared to those without an education level (OR = 2.43, 95% CI = 1.30–6.20). Additionally, single individuals were three times more likely to have a positive perception than their married counterparts (OR = 3.39, 95% CI = 1.28–9.09). Vaccinated individuals had twice the odds of positive perception than non-vaccinated individuals (OR = 2.89, 95% CI = 1.01–8.89). Those receiving information from government health institutions were three times more likely to have a positive perception than those who received the information from friends (OR = 3.19, 95% CI = 1.02–12.7). Moreover, employed participants were four times more likely to have a positive perception non-employed individuals (OR = 4.21, 95% CI = 1.48–13.6). Besides, gender and COVID-19 diagnosis did not significantly correlate with positive COVID-19 vaccine perception.

**Conclusion:**

The results indicate that the general public in Rwanda has good knowledge, positive attitudes, and a positive perception toward the COVID-19 vaccination, however, some of the participants had some misconceptions towards COVID-19. The findings of this study will be valuable for policymakers and healthcare authorities working to improve vaccination rates.

**Supplementary Information:**

The online version contains supplementary material available at 10.1186/s12889-024-19082-9.

## Introduction

With the emergence and global spread of the 2019 novel coronavirus (2019-nCoV) or severe acute respiratory syndrome coronavirus 2 (SARS-CoV-2), the world has faced a public health crisis [1]. The World Health Organization (WHO) has reported 753,001,888 confirmed cases of coronavirus disease 2019 (COVID-19), with 6,807,572 deaths, illustrating the immense burden of COVID-19 on the global population [2]. According to the WHO, Rwanda has also been significantly affected, reporting 133,090 confirmed cases and 1,468 deaths [2, 3]. The pandemic has profoundly impacted individuals and communities worldwide, shaping their perspectives on COVID-19 vaccines [4]. Individuals with favorable attitudes and a high degree of awareness about COVID-19 vaccination are likelier to follow public health guidelines such as mask use and social distancing while waiting for the vaccine [5]. Vaccine hesitancy is described as hesitation or outright refusal to get a vaccine [6], and different factors, such as regional differences, time variations, and vaccine-specific traits, contribute to the complexity of vaccine hesitancy and this have been linked to the low COVID-19 vaccinations rates in some countries [7]. Similar complications have arisen with other contagious illnesses; for example, measles, long believed to be extinct, is suddenly making a dramatic comeback. On the other hand, it has been found that the increased number of COVID-19 cases made the widespread unwillingness to get the vaccination all the more important from a public health perspective [8]. For instance, a study investigating vaccine hesitancy among adult Africans found that 33% of respondents stated they were unlikely to receive the vaccine, while 15% remained undecided; in addition, the study also highlighted the association between vaccine hesitancy and socio-demographic factors such as age, gender, education, and sources of information [9]. Furthermore, another study conducted among healthcare workers in Ethiopia showed that a significant proportion demonstrated commendable knowledge, positive attitudes, and willingness to receive the COVID-19 vaccine; however, vaccine refusal was primarily linked to negative attitudes and suboptimal perceptions, with concerns about safety and efficacy frequently cited [10]. Additionally, a separate study found that although vaccine hesitancy in Kenya is relatively lower than in other countries, however, this issue remains a persistent challenge [11]. The level of health knowledge among participants is a critical factor that affects their acceptance of health measures, and a well-informed public is better equipped to respond to pandemics such as COVID-19 by learning about the disease, vaccination options, the advantages and risks associated with these measures [12]; and two main cognitive factors heavily influence the rate of COVID-19 vaccination: attitude and perception [5, 12, 13].

Following the footsteps of other countries, Rwanda has initiated its vaccination program, reducing COVID-19 cases and according to reports from the Rwanda Biomedical Center (RBC), over 80% of the Rwandan population has been vaccinated [14]. Despite the efforts put into vaccination campaigns and numerous research studies conducted in Africa and worldwide, there is currently a lack of published literature specifically focusing on Rwandans’ knowledge, attitudes, and perceptions towards COVID-19 vaccines. For the most part, KAP surveys are useful for implementing successful public health interventions since they reveal knowledge gaps and behavioural patterns of the general population based on their socio-demographics [15]. Therefore, this study examined the Rwandan population’s knowledge, attitudes, and perceptions towards COVID-19 vaccinations.

## Methods

### Study design, setting, and population

The survey utilized a cross-sectional design. A semi-structured online questionnaire was created using Google Forms and attached to the respondents’ permission form. The survey was conducted in Rwanda from February 11th to February 17th, 2023. The study population consisted of individuals aged 18 years and older residing in Rwanda’s four provinces and Kigali City. The single population proportion formula was used to determine the sample size for this study with the following assumptions: a 95% confidence interval (CI), a 5% margin of error, and a previously reported vaccine perception estimate of 40% (12). Finally, it had 370 participants in total. Simple random sampling methods were used to select the individuals with sufficient contact information in Health Center family books. The Health Center family books are comprehensive records containing information about individuals registered with each health centers. These records typically include demographic details such as name, age, gender, address, and sometimes additional health-related information like medical history, immunizations, and family medical records.

### Measures

The questionnaires were designed based on reviewing literature published in reputable journals [16–18]. The online self-reported and respondent-friendly questionnaire developed for this study contained questions assessing socio-demographics and other parts that mainly focused on knowledge, attitude, and vaccine perception. The socio-demographic variables included age, gender, marital status, level of education, occupation, location, COVID-19 diagnosis Status, source of information, and COVID-19 vaccination status. The knowledge section comprised statements to assess knowledge of the COVID-19 vaccine. There was also an attitude and vaccine perception section that contained attitudes toward COVID-19 vaccinations and preventive measures, as well as the immune response against COVID-19 statements. This research begins with an inquiry regarding the ability of vaccines to generate an immune response against COVID-19. Then, the data were collected through various channels, including emails, WhatsApp, and other social media platforms. The questionnaire is available in Supplementary Materials File S1.

Questions were adapted previous published article on vaccinations [19–26]. The knowledge section consisted of five questions, each with three possible replies (“Yes,” “No,” and “Don’t know”). ‘Yes’ was scored 1, while ‘No/Don’t know’ replies were 0. The total score was calculated by summing the raw scores of the five elements, which ranged from 0 to 5; participants were categorized as having either good or poor knowledge based on their aggregate score (mean and above = good, below mean = poor) [27]. The attitude section consisted of 6 statements, each offering three possible replies: “Agree,” “Disagree,” and “Neutral.” The score for ‘agree’ was 1, while ‘disagree’ and ‘neutral’ replies were scored as 0. The total score was 6, calculated by summing the raw scores of the 6 elements and participants were categorized as having positive or negative attitudes based on their aggregate score (mean and above = positive, below mean = negative) [28]. Perception was evaluated through a single statement regarding belief in the vaccines’ ability to produce an immune response. Respondents who answered affirmatively were classified as having a positive perception, while those who responded negatively were categorized as having a negative perception [24]; the study’s main outcome was the perception of the COVID-19 vaccine. Before the data collection. The questionnaires were pretested on 20 individuals who were later omitted from the study.

### Analysis

The data were analyzed using R software version 4.4. Counts and percentages were used to represent categorical variables. The Fisher Exact and Chi-square tests compared different groups’ COVID-19 knowledge, attitudes, and perceptions. The binary logistic regression analysis was used to assess the determinants of COVID-19 vaccine perception. In the initial analysis, independent variables demonstrating a P-value of ≤ 0.25 were selected for inclusion in the final multivariable model for further investigation. To ensure the robustness of the findings, potential multicollinearity among variables was evaluated using the variance inflation factor (VIF), with a median VIF threshold of < 5 [29].The adequacy of the model fit was assessed using the Hosmer and Lemeshow goodness of fit test, where a p-value ≥ 0.05 was considered indicative of satisfactory fit [30]. The strength of the relationship and its statistical significance were gauged using Adjusted Odds Ratios (AOR) along with 95% confidence intervals (CI), with a significance level set at *P* ≤ 0.05 [31].

### Ethical consideration

The study on COVID-19 vaccine knowledge, attitude, and perception in Rwanda followed ethical standards, ensuring the protection of participants’ rights, confidentiality, and well-being throughout the research process. This study was approved by the ethical approval of the Institutional Ethics Committee of the School of Public Health, Cheeloo College of Medicine, Shandong University (LL20230101). All participants provided informed consent before taking part in the study, and their data were handled with strict confidentiality. The research team-maintained transparency in reporting the findings and acknowledged the support received from various funding sources. The study’s ethical considerations underscored the importance of upholding integrity and respect for participants, contributing to the credibility and reliability of the research outcomes.

## Results

### Socio-demographic characteristics

Three hundred seventy participants filled in the survey and were recruited in this study. Of this study’s participants (see Table 1), 45.4% were between the ages of 30 and 39. Over half of the participants (50.5%) were females. Respondents had various education levels, with the majority having the university level (59.7%), followed by secondary (23.5%), no-education level (9.5%), and primary level (7.3%). Most respondents were married (64.3%), and only 35.7% were single. The largest group of participants came from the Northern province (48.1%), followed by Kigali City (34.9%). The Southern province accounted for 7.6% of participants, while the Eastern province accounted for 4.9%. The Western province had the lowest representation, with only 4.5%. Regarding accessing vaccine information, most respondents preferred to get the vaccine information from Government health institutions (68%). Among the respondents, 53.5% were diagnosed with COVID-19, 93% had received the COVID-19 vaccine, and 77.3% were employed.

**Table 1:** Socio-demographic characteristics

Characteristic	*n* = 370	*n* (%)
**Age**		
18–29	95	25.7
30–39	168	45.4
40–49	37	10.0
50–59	23	6.2
60 and above	47	12.7
**Gender**		
Female	183	49.5
Male	187	50.5
**Education level**		
None	35	9.5
Primary	27	7.3
Secondary	87	23.5
University	221	59.7
**Marital status**		
Married	238	63.3
Single	132	35.7
**Location**		
Eastern Province	18	4.9
Kigali city	129	34.9
Northern Province	178	48.1
Southern Province	28	7.6
Western Province	17	4.5
**COVID-19 vaccination status**		
Vaccinated	344	93.0
Non-vaccinated	26	7.0
**COVID-19 information sources**		
Friends	18	4.9
Government health institutions	253	68.4
Other sources	24	6.5
Social media	75	20.2
**Occupation**		
Employed	286	77.3
Non-employed	51	13.8
Student	33	8.9
**COVID-19 diagnosis**		
Not diagnosed	172	46.5
Diagnosed	198	53.5

## Knowledge of participants towards COVID-19 vaccines

85% of the participants had good knowledge, while only 15% had poor knowledge about COVID-19 vaccines **(**Supplementary Table 1). 85.6% of respondents knew of the COVID-19 vaccination, and 58.6% knew its efficacy. Additionally, a significant number of participants were unaware of the dangers of overdosing on the vaccines, whereas 46.5% of respondents were aware of the risks. Furthermore, 82.2% of respondents knew that vaccines might lead to allergic responses, while 86.2% recognized that vaccination could raise the risk of autoimmune illnesses Table 2. The average knowledge was 3.58(SD:1.24) (Supplementary Table 1).

**Table 2:** Distribution of each knowledge questions

Statements	Total *n* (%)
**Do you know about the COVID-19 vaccine?**	
Yes	313 (85.6)
No	21 (5.7)
I don’t know	36 (9.7)
**Do you know about the effectiveness of COVID-19 vaccine?**	
Yes	217 (58.6)
No	95 (25.7)
I don’t know	58 (16.7)
**Do you know that it is dangerous to use overdose vaccines?**	
Yes	172 (46.5)
No	178 (48.1)
I don’t know	20 (5.4)
**Does vaccination increase allergic reactions?**	
Yes	304 (82.2)
No	8 (2.2)
I don’t know	58 (15.6)
**Does vaccination increase autoimmune diseases?**	
Yes	319 (86.2)
No	10 (2.7)
I don’t know	41 (11.1)

### Attitude and perception of study participants toward the COVID-19 vaccines

A total of 282 participants in the study reported a positive perception, while only 88 participants reported a negative perception and those with a positive perception believed that COVID-19 vaccines stimulate an immune response against COVID-19. In this study, 86% of the participants held a positive attitude, while only 14% had a negative attitude towards COVID-19 vaccines. The average attitude score was 4.3 (SD: 1.7) (see Supplementary Table 2). Conversely, 83.2% of the respondents agreed that individuals who have recovered from COVID-19 do not need vaccination. Additionally, 76.5% believed that COVID-19 played a significant role in reducing the number of COVID-19 cases. Importantly, 67.6% of the study participants disagreed that people should stop practicing precautions such as wearing masks, maintaining social distancing, and practicing proper hand hygiene after receiving the COVID-19 vaccine. As a result of the pandemic, some respondents indicated a change in their attitude towards vaccination. However, 86.5% still held a positive attitude towards vaccinating children under 15. Moreover, 83.0% of the participants in this study agreed that, despite not everyone being vaccinated, the pandemic would soon end (Table 3).

**Table 3:** Attitude and perception of study participants toward the COVID-19 vaccine

Statements	Total *n* (%)
**Do you believe that the vaccines produce an immune response against COVID-19?**	
Negative vaccine perception	88 (23.8)
Positive vaccine perception	282 (76.2)
**Do you agree that vaccines played important roles in the reduction of COVID-19?**	
Agree	283 (76.5)
Disagree	40 (10.8)
Neutral	47 (12.7)
**Do you agree that people who have had COVID-19 and recovered do not need to get vaccinated?**	
Agree	308 (83.2)
Disagree	21 (5.7)
Neutral	41 (11.1)
**Stop practicing precautions such as masking, social distancing, and hand hygiene after receiving the COVID-19 vaccine.**	
Agree	73 (19.7)
Disagree	250 (67.6)
Neutral	47 (12.7)
**The COVID-19 pandemic changed my approach to vaccination.**	
Agree	316 (85.4)
Disagree	10 (2.7)
Neutral	44 (11.9)
**Kids under 15 years should get vaccinated.**	
Agree	320 (86.5)
Disagree	10 (2.7)
Neutral	40 (10.8)
**Although we are unable to vaccinate everyone, the epidemic will stop soon.**	
Agree	307 (83.0)
Disagree	9 (2.4)
Neutral	54 (14.6)

### Associations between socio-demographic characteristics, attitudes, knowledge, and perceptions towards COVID-19 vaccines

As indicated in Table 4, a significant correlation was observed between a positive perception of the COVID-19 vaccine and several variables, including age category (P value < 0.001), education level (P value < 0.001), location (P value < 0.001), information source (P value < 0.001), and COVID-19 vaccination status (P value < 0.001). Furthermore, our findings revealed that a considerable proportion of participants exhibited a positive attitude and good knowledge and showed a statistical significance with perception regarding the COVID-19 vaccine (P value < 0.001) (Table 5).

**Table 4:** Associations between socio-demographic characteristics and positive perceptions (*n* = 370), *p < 0.05

Variables	*n* (%)	Negative vaccine perception; (%)	Positive vaccine perception; *n* (%)	*P* value
Age				< 0.001*
18–29	95 (25.7)	25 (28.4)	70 (24.8)	
30–39	168 (45.4)	23 (26.1)	145 (51.4)	
40–49	37 (10.0)	16 (18.2)	21 (7.4)	
50–59	23 (6.2)	11 (12.5)	12 (4.3)	
60 and above	47 (12.7)	13 (14.8)	34 (12.1)	
Gender				0.5
Female	183 (49.5)	46 (52.3)	137 (48.6)	
Male	187 (50.5)	42 (47.7)	145 (51.4)	
Education level				< 0.001*
None	35 (9.5)	14 (15.9)	21 (7.4)	
Primary	27 (7.3)	13 (14.8)	14 (5.0)	
Secondary	87 (23.5)	16 (18.2)	71 (25.2)	
University	221 (59.7)	45 (51.1)	176 (62.4)	
Marital status				0.3
Married	238 (64.3)	61 (69.3)	177 (62.8)	
Single	132 (35.7)	27 (30.7)	105 (37.2)	
Location				< 0.001*
Eastern Province	18 (4.9)	2 (2.3)	16 (5.7)	
Kigali city	129 (34.9)	33 (37.5)	96 (34.0)	
Northern Province	178 (48.1)	35 (39.8)	143 (50.7)	
Southern Province	28 (7.6%)	16 (18.2%)	12 (4.3%)	
Western Province	17 (4.6)	2 (2.3)	15 (5.3)	
Vaccination status				< 0.001*
Not vaccinated	26 (7.0)	14 (15.9)	12 (4.3)	
Vaccinated	344 (93.0)	74 (84.1)	270 (95.7)	
Information sources				< 0.001*
Friends	18 (4.9)	7 (8.0)	11 (3.9)	
Government health institutions	253 (68.4)	44 (50.0)	209 (74.1)	
other sources	24 (6.5)	13 (14.8)	11 (3.9)	
Social media	75 (20.3)	24 (27.3)	51 (18.1)	
Occupation				0.3
Employed	286 (77.3)	68 (77.3)	218 (77.3)	
Non-employed	51 (13.8)	9 (10.2)	42 (14.9)	
Student	33 (8.9)	11 (12.5)	22 (7.8)	
Covid diagnosis				0.3
Not diagnosed	172 (46.5)	45 (51.1)	127 (45.0)	
Diagnosed	198 (53.5)	43 (48.9)	155 (55.0)	

**Table 5:** Associations between attitude, knowledge, and positive perceptions (*n* = 370), *p < 0.05

	Total *n* (%)	Negative perception *n* (%)	Positive perception *n* (%)	*P*-value
**Attitude status**				< 0.001
Negative	60(16.2)	28 (7.6)	32 (8.6)	
Positive	310(83.8)	60 (16)	250 (68)	
**Knowledge status**				< 0.001
Poor	55 (14.9)	25 (6.8)	30 (8.1)	
Good	315(85.1)	63 (17)	252 (68)	

### Positive COVID-19 perception predictors towards COVID-19 present vaccines

The univariate analysis found that belonging to the group aged 30–39 and 50–59, having university and secondary level, being single, being vaccinated, and getting information from government health institutions were associated with positive perceptions of COVID-19 vaccines (*P* < 0.05). On the other side, our multivariate analysis revealed that the age group of 30–39, having a university education level, being single, being vaccinated, getting information from government health institutions, and being non-employed were the only variables associated with a positive perception of COVID-19 vaccines. Participants belonging to the group aged 30–39 have 1.39 higher odds compared to other age groups (OR = 1.39, 95% CI = 1.08–3.24). Participants with a university education level had double the odds of having positive COVID-19 vaccine perception compared to those without education level (OR = 2.43, 95% CI = 1.30–6.20). Additionally, single participants had three times higher odds of having a positive COVID-19 vaccine perception than married participants (OR = 3.39, 95% CI = 1.28–9.09). Moreover, vaccinated people had double odds of having positive COVID-19 vaccine perception compared to those who did not receive the vaccines (OR = 2.89, 95% CI = 1.01–8.89). We also found that participants who got the information from the government health institution had three times higher odds of having a positive COVID-19 vaccine perception than those who got the information from their friends (OR = 3.19, 95% CI = 1.02–12.7). Also, employed participants had four times higher odds of having a positive COVID-19 vaccine perception than non-employed participants (OR = 4.21, 95% CI = 1.48–13.6). Gender and COVID-19 diagnosis had no association with positive COVID-19 vaccine perception (Table 6).

**Table 6:** Univariable and multivariable analysis of determinants factors for positive perception of COVID-19 vaccination. * *P* < 0.05

Variable	Univariate analysis				Multivariate analysis		
	*n*	OR^1^	95% CI^1^	*P* value	OR^1^	95% CI^1^	*P* value
**Age**	370						
18–29		1.00	—		—	—	
30–39		2.25	1.19, 4.27	0.012*	1.39	1.08, 3.24	0.015*
40–49		0.47	0.21, 1.04	0.062	0.30	0.09, 0.93	0.07
50–59		0.39	0.15, 1.00	0.049*	0.23	0.07, 0.75	0.4
60 and above		0.93	0.43, 2.09	0.86	0.62	0.21, 1.88	0.78
**Gender**	370						
Female		1.00	—		—	—	
Male		1.16	0.72, 1.88	0.55	1.83	0.39, 7.04	0.4
**Education level**	370						
None		1.00	—		—	—	
Primary		0.72	0.26, 1.98	0.52	0.83	0.23, 3.02	0.8
Secondary		2.96	1.24, 7.10	0.014*	1.70	0.84, 6.90	0.10
University		2.61	1.21, 5.50	0.012*	2.43	1.30, 6.20	0.015*
**Marital status**	370						
Married		1.00	—		—	—	
Single		1.34	0.81, 2.27	0.246	3.39	1.28, 9.09	0.014*
**Location**	370						
Eastern Province		1.00	—		—	—	
Kigali city		0.36	0.06, 1.37	0.19	0.36	0.05, 1.70	0.2
Northern Province		0.51	0.08, 1.91	0.38	0.57	0.07, 2.82	0.5
Southern Province		0.09	0.01, 0.41	0.005*	0.11	0.01, 0.76	**0.036**
Western Province		0.94	0.10, 8.64	0.95	1.45	0.12, 18.5	0.8
**Vaccination status**	370						
Not vaccinated		1.00	—		—	—	
Vaccinated		4.26	1.89, 9.75	< 0.001*	2.89	1.01, 8.36	0.047*
**Information sources**	370						
Friends		1.00	—		—	—	
Government health institutions		3.02	1.06, 8.12	0.030*	3.19	1.02, 12.7	0.038*
other sources		0.54	0.15, 1.84	0.33	0.32	0.06, 1.63	0.2
Social media		1.35	0.45, 3.88	0.58	1.00	0.24, 4.16	> 0.9
**Occupation**	370						
Non-employed		1.00	—		—	—	
Employed		1.46	0.70, 3.33	0.34	4.21	1.48, 13.6	0.011*
Student		0.62	0.29, 1.40	0.23	0.64	0.24, 1.83	0.4
**Covid diagnosis**	370						
No		1.00	—		—	—	
Yes		1.28	0.79, 2.07	0.32	1.22	0.28, 6.33	0.8

^1^OR = Odds Ratio, CI = Confidence Interval

## Discussions

This is the first study to assess the knowledge, attitude, and perception of COVID-19 vaccinations among the Rwandan population and to reveal the factors that most strongly influence vaccine perception. Compared to studies in Ethiopia [17, 25] and Uganda [26], our results showed that a larger percentage of respondents had good knowledge, positive attitudes and perception of COVID-19 vaccinations. Furthermore, most participants knew the vaccination’s effectiveness and recognized potential risks like allergic responses and autoimmune illnesses. Our results agreed with studies conducted in Ethiopia [32] and Somalia [33]; this may be attributed to the diligent efforts exerted by the Rwandan government in promoting awareness and prevention measures about the COVID-19 pandemic.

Many participants had a positive attitude towards COVID-19 vaccinations, supporting their ability to stimulate an immune response against the virus. This finding is consistent with the research done in Tanzania with pregnant women [34] and another one conducted in Kenya [35]. Furthermore, a substantial portion of the participants also exhibited pessimistic perspectives, such as the notion that individuals who have recuperated from COVID-19 do not necessitate vaccination. In contrast to our findings, a study conducted in Kenya found quite different findings, stating that many participants believed receiving the vaccine was necessary, even if they had already recovered from COVID-19 [36], and misconception reported by our study, maybe attributed to the lack of comprehensive knowledge and information regarding COVID-19 and Its vaccination during the initial phases of the pandemic.

Furthermore, our results aligned with studies conducted in Zimbabwe [37] and another in West African countries [38], which reported that factors such as age, education level, location, vaccination, and source of information were significantly associated with positive perception towards COVID-19 vaccines, these results imply that it is always important to pay attention to these connections to critically develop customized public health strategies to reduce vaccination hesitancy and increase universal adoption of COVID-19 vaccines. Additionally, our study also revealed that participants who believed in the importance of vaccines in reducing COVID-19 cases and continued to practice precautions post-vaccination, who had positive attitudes and good knowledge, were associated with a positive perception of the COVID-19 vaccine, emphasizing the importance of educational campaigns related to COVID-19 vaccines, our results were in line with those from a study conducted in Nigeria [39]. Our research findings also indicated a shift in participants’ attitudes towards vaccination due to the pandemic, with a majority still supporting the vaccination of children under 15 and believing in the eventual end of the pandemic despite not everyone being vaccinated; our results were comparatively the same to the ones reported by a study conducted in Turkey [24], however, even though we got such good results a recent study reported different results reporting that their study’s participants did not believe that their children need to be vaccinated [40]. These evolving attitudes reflect the dynamic nature of public opinion towards vaccination in the face of a global health crisis. Furthermore, there was a statistical significance between attitudes, knowledge, and perceptions regarding the COVID-19 vaccine, and this was further supported by a study conducted by M Kearney et al. [41], these results demonstrate the need for identifying and resolving the factors influencing knowledge, attitudes, and perceptions of the COVID-19 vaccines, as well as knowledge of these components, to properly promote vaccine acceptance, respond to concerns, and support public health programs.

Respondents between 30 and 39 were found to be more likely to have positive perceptions of COVID-19 vaccines, these findings align with those of a study conducted in multiple African countries, which found that South Africans in this age group had more favorable perceptions towards COVID-19 vaccines than participants of other age groups [42]. Moreover, single participants were more likely to have positive vaccine perceptions compared to married participants; a study conducted in Singapore reported nearly the same results [43, 44]; however, in their multivariate analysis, they discovered that this association was non-significant [45, 46], our analysis found that males were more likely to have a positive perception toward COVID-19, however, there was no significantly impact shaping COVID-19 vaccine perceptions. This deviation could be attributed to shifting gender roles and the shared responsibility of healthcare decision-making between genders, suggesting a lack of apparent gender-based disparities in vaccine hesitancy.

Furthermore, university education level emerged as a significant predictor associated with positive COVID-19 vaccine perceptions, with higher education levels showing a greater belief in vaccine safety and effectiveness. This finding is consistent with studies conducted in Zambia [47] and Nigeria [48] that reported a positive relationship between knowledge and COVID-19 positive perception, with education being a significant determinant. These results suggest that education plays an essential role in overcoming such pandemic, as educated persons are more likely to assess key circumstances and, as a result, respond constructively. Participants who have been vaccinated were more inclined towards positive vaccine perceptions, supported by a study conducted by Oluseyi et al. [49]. Additionally, obtaining information from government health institutions was associated with a more favorable perception of COVID-19 vaccines, reflecting the trust in official sources for reliable vaccine-related information. In contrast, social media and word-of-mouth channels were perceived as less trustworthy sources, which may contribute to the spread of misconceptions about vaccines [50]. Furthermore, employed participants tended to exhibit more positive perceptions than unemployed individuals, aligning with previous research emphasizing the positive correlation between employment status and vaccine acceptance [51, 52].

The government put much effort into explaining to its people the benefits of getting COVID-19 vaccines, specifically from the high-ranked government institutions to the lowest-ranked local government institutions [53]. Religions in Rwanda play an essential role in different government campaigns that help improve the well-being of the Rwandan society; during COVID-19, this religious sector quickly understood their part and supported the government in explaining the importance to their church members of getting COVID-19 vaccines; this increased COVID-19 perception. Rwanda responded promptly to the outbreak. The government imposed a statewide lockdown in early March 2020, blocking borders and restricting foreign travel [54]. These precautions aided in preventing the virus’s fast spread and gave time for the healthcare system to be strengthened [55]. Rwanda took a proactive approach to testing and contact tracing. The government quickly increased its testing capacity and built a solid contact tracking system. This aggressive strategy enabled the detection and isolation of sick individuals, minimizing the virus’s spread within communities [55]. The Rwandan government displayed outstanding leadership and a well-coordinated pandemic response. Clear communication, good public health messaging, and the participation of community leaders all aided in mobilizing the populace and gaining their cooperation in following preventative measures [56]. On the other hand, the Rwandan government emphasized public engagement, awareness campaigns, and collaboration with community leaders to promote vaccine uptake and build trust [57]. Overall, the facts mentioned above played an essential role in the high COVID-19 vaccine perception, positive attitude, and good knowledge that our study reported. The results of this study have the potential to aid health officials in their pursuit of high vaccine coverage goals by providing them with more accurate and up-to-date information and facilitating better communication.

### Strengths and limitations

This research sheds light on the knowledge, attitude status, and perception of COVID-19 vaccines in Rwanda. This information is expected to assist Rwandan health authorities in formulating appropriate measures to increase the rates of COVID-19 vaccinations and establish best practices for future epidemics. However, our study also has some limitations because no appropriate questionnaire was available to the Rwandan population throughout the research period; we were first unable to use a previously validated questionnaire. Secondly, some people may not be able to participate in the survey because they do not have a computer, phone, or other device that can connect to the internet or social media sites like Facebook, WhatsApp, and other online platforms. Thirdly, there is a chance that the sample is skewed toward those with more education, which might lead to inflated findings since people with more education are more likely to have a positive perception of COVID-19 vaccinations and to know more about them. These constraints provide possibilities for more study.

## Conclusions

Our study’s findings provide a thorough a recap of participants’ sociodemographic features, attitudes, knowledge, and views of COVID-19 vaccinations. The interdependence of these elements emphasizes the need of customized public health measures that take demographic diversity, educational outreach, and individual beliefs into account when determining vaccine acceptability. Understanding the many factors influencing vaccination views allows governments and healthcare professionals to customize approaches to enhance vaccine uptake, eliminate rumors, and encourage a collaborative approach to tackling the COVID-19 pandemic. Our study’s results are intended to help policymakers and health professionals develop evidence-based approaches that will increase confidence in COVID-19 vaccinations, not just in Rwanda, but also in communities throughout the globe.

### Electronic supplementary material

Below is the link to the electronic supplementary material.

Supplementary Material 1

Supplementary Material 2

Supplementary Material 3

## Data Availability

The datasets analyzed during the current study are not publicly available due to an ongoing project, but are available from the corresponding author on reasonable request.
