# Supplementary material for: Knowledge, attitude, and perception towards COVID-19 vaccinations among the adults in Rwanda: a cross-sectional study
Source: BMC Public Health. 2024 Jul 17;24:1919. doi: 10.1186/s12889-024-19082-9 (PMC11256467; doi:10.1186/s12889-024-19082-9)
Supplement: Supplementary file 1 — Supplementary Material 1 [file 12889_2024_19082_MOESM1_ESM.docx]

**Supplementary files**

**S1: Questionnaire**

**Knowledge, Attitudes, and Perception about COVID-19 Vaccination among the adult population: A Cross-Sectional Study in Rwanda**

We are conducting a survey entitled "Knowledge, Attitudes, and Perception towards COVID-19 Vaccination among Adult Population: A Cross-Sectional Study in Rwanda". The study's findings will reflect people's knowledge, attitudes, and perceptions of COVID-19 vaccines. The survey might last between 2 minutes. You will not be asked for any personal information in the survey. Your personal information will be kept strictly confidential. You have the right to participate or refuse, and you may withdraw from replying at any moment throughout your involvement. The research will not benefit you financially or otherwise, but policymakers may consider the findings and lead to the implementation of COVID-19 immunizations in Rwanda. Thank you for taking the time to participate.

**Contact information: Abakundana Nsenga Ariston Gabriel email:** [**abakundanagaby@gmail.com**](mailto:abakundanagaby@gmail.com)

**tell :(+250)785303885**

Consent

1. Are you willing to participate in this survey?

- Yes
- No

**Section 1: Social-demographic characteristics**

**2. 1. Age**

- 18-29
- 30-39
- 40-49
- 50-59
- 60 and above

**3. 2. Gender**

- Female
- Male

**4. 3. Education level**

- none
- Primary
- Secondary
- University

**5. 4. Occupation status**

- Employed
- Non-employed
- Student

**6. 5. Marital status**

- Married
- Single

**7. 6. Location**

- Kigali city
- Northern province
- Southern province
- Western province
- Eastern province

**8. 7. Have you ever been diagnosed with COVID -19?**

- Yes
- No

**9. 8. Have you ever been vaccinated?**

- Yes
- No

**10. 9. Which of the following sources will you choose to derive Covid-19 vaccine-related information**

- Social media
- Friends
- Government health institutions
- Other sources

**Section 2: Knowledge**

**11. 1. Do you know about the COVID-19 vaccine?**

- Yes
- No
- I don’t know

**12. 2. Do you know about the effectiveness of COVID-19 vaccine?**

- Yes
- No
- I don’t know

**13. 3. Do you know that it is dangerous to use overdose vaccines?**

- Yes
- No
- I don’t know

**14. 4. Does vaccination increase allergic reactions?**

- Yes
- No
- I don’t know

**15. 5. Does vaccination increase autoimmune diseases?**

- Yes
- No
- I don’t know

**Section 3: Attitude and vaccine perception**

**16. 1. Do you believe that the vaccines produce an immune response against COVID-19**

**?**

- Yes
- No

**17. 2. Do you agree that vaccines played important roles in reducing COVID-19 cases?**

- Agree
- Disagree
- Neutral

**18. 3. Do you agree that people who have had COVID-19 and recovered no need to get vaccinated?**

- Agree
- Disagree
- Neutral

**19. 4. Stop practicing precautions such as masking, social distancing, and hand hygiene after receiving COVID-19 vaccine.**

- Agree
- Disagree
- Neutral

**20. 5. COVID-19 pandemic changed my approach to vaccination**

- Agree
- Disagree
- Neutral

**21. 6.Kids under 15 years should get vaccinated?**

- Agree
- Disagree
- Neutral

**22. 7. Despite the fact that we are unable to vaccine everyone, the epidemic will stop soon.**

- Agree
- Disagree
- Neutral
