# Supplementary material for: Knowledge, attitude, and perception towards COVID-19 vaccinations among the adults in Rwanda: a cross-sectional study
Source: BMC Public Health. 2024 Jul 17;24:1919. doi: 10.1186/s12889-024-19082-9 (PMC11256467; doi:10.1186/s12889-024-19082-9)
Supplement: Supplementary file 2 — Supplementary Material 2 [file 12889_2024_19082_MOESM2_ESM.docx]

**Supplementary table 2: attitude score and status**

|  | **n= 370** |
| --- | --- |
| **Attitude Score(M±SD)** | 4.3±1.7 |
| **Attitude status** |  |
| Negative | 60 (16%) |
| Positive | 310 (84%) |
|  | |
