# Supplementary material for: Knowledge, attitude, and perception towards COVID-19 vaccinations among the adults in Rwanda: a cross-sectional study
Source: BMC Public Health. 2024 Jul 17;24:1919. doi: 10.1186/s12889-024-19082-9 (PMC11256467; doi:10.1186/s12889-024-19082-9)
Supplement: Supplementary file 3 — Supplementary Material 3 [file 12889_2024_19082_MOESM3_ESM.docx]

**Supplementary table 1: Knowledge score and level**

|  | **n= 370** |
| --- | --- |
| **Knowledge score (**Mean and SD) | 3.5±1.2 |
| **Knowledge level** |  |
| poor | 55 (15%) |
| good | 315 (85%) |
|  | |
